# *In vivo* evidence for homo- and heterodimeric interactions of *Arabidopsis thaliana* dehydrins AtCOR47, AtERD10, and AtRAB18

**DOI:** 10.1038/s41598-017-15986-2

**Published:** 2017-12-06

**Authors:** Itzell E. Hernández-Sánchez, Israel Maruri-López, Steffen P. Graether, Juan F. Jiménez-Bremont

**Affiliations:** 1 Laboratorio de Biología Molecular de Hongos y Plantas, División de Biología Molecular, Instituto Potosino de Investigación Científica y Tecnológica AC, San Luis Potosí, Mexico; 2 Department of Molecular and Cellular Biology, University of Guelph, Guelph, ON Canada

## Abstract

Dehydrins (DHNs) are intrinsically disordered proteins that play central roles in plant abiotic stress responses; however, how they work remains unclear. Herein, we report the *in planta* subcellular localization of *Arabidopsis thaliana* DHNs AtCOR47, AtERD10, and AtRAB18 through GFP translational fusions. To explore the dimerization ability of the Arabidopsis acidic DHNs AtCOR47 and AtERD10, we conducted an *in planta* DHN binding assay using the Bimolecular Fluorescence Complementation (BiFC) technique. Our analyses revealed homodimeric interactions for AtCOR47 and AtERD10; interestingly, heterodimeric associations also occurred with these DHNs, and these interactions were observed in the cytosol of tobacco cells. Furthermore, we evaluated whether Arabidopsis basic DHNs, such as AtRAB18, could also interact with itself and/or with AtCOR47 and AtERD10 in the BiFC system. Our data revealed homodimeric RAB18 complexes in the nucleus and cytosol, while heterodimeric associations between AtRAB18 and acidic DHNs occurred only in the cytosol. Finally, we demonstrated the presence of heterodimeric complexes among Arabidopsis AtCOR47, AtERD10, and AtRAB18 DHNs with their acidic ortholog the OpsDHN1 from *Opuntia streptacantha*; these heterodimeric interactions showed different subcellular distributions. Our results guide DHN research toward a new scenario where DHN/DHN oligomerization could be explored as a part of their molecular mechanism.

## Introduction

Global climate change is a fact; this phenomenon is accompanied by environmental stresses factors like drought, salinity, and extreme temperatures with which plants must deal with more often in order to survive. To withstand stress, plants have evolved physiological and molecular responses involving changes in their transcriptome, proteome, and metabolome^1^. Dehydrins (DHNs) are plant-specific proteins that accumulate during the abiotic stress that cause cellular dehydration, such as drought, salinity, freezing, or by treatment with the phytohormone ABA^2^. Transcriptional and proteomic studies of abiotic tolerance in several plant species have demonstrated that DHNs transcripts and protein levels increase in tolerant varieties^3–5^. In addition, transgenic studies have widely demonstrated the positive effect of DHNs expression and accumulation in order to survive to changing conditions^6–8^. Based on *in vitro* experiments it has been hypothesized that this effect relies on cryoprotective and chaperone activity, as well as metal and reactive oxygen species scavenging functions; however, not all DHNs exhibit all of these functions^2^.

According to their expression patterns and physicochemical properties, DHNs can be classified into two groups, 2a and 2b^9^. Proteins that belong to group 2a are preferentially expressed during the late embryogenesis stage, and are proteins with basic or neutral isoelectric points. Members of group 2b are associated with cold tolerance, and do not typically accumulate during the late embryogenesis stage. The 2b members also contain a large proportion of acidic residues^10^. In the model plant *Arabidopsis thaliana* genome, until now 10 DHNs genes have been annotated^11^; the DHN acidic group contains six genes At1g20440 (AtCOR47), At1g20450 (AtERD10), At1g76180 (ERD14), At2g21490, At4g39130, and At4g38410, and the remaining four genes are part of basic or neutral DHN group, At1g54410 (AtHIRD11), At3g50970 (XERO2), At3g50980 (XERO1) and At5g66400 (AtRAB18)^11^.

In *A*. *thaliana*, AtERD10 and AtCOR47 are the principal DHNs that accumulate in response to low temperature, contributing to the cold stress response^12^. *In vitro* functions have been established for *A*. *thaliana* acidic DHNs AtCOR47 and AtERD10, such as ion and water-binding, cryoprotective activity, thylakoid membrane-binding, and metal-binding^13–15^. In particular, chaperone activity has been reported for ERD10^16^. The over-expression of AtCOR47 and AtERD10 correlated with an improved *A*. *thaliana* cold stress tolerance under low-temperature conditions^12^. In addition, the *erd10* T-DNA insertional mutant showed reduced tolerance to drought, cold stress and a reduction on seed germination^17^. The Arabidopsis basic DHN, AtRAB18, is also up-regulated and accumulates under low temperatures, drought, salinity, and ABA, suggesting that it is involved in these abiotic stress responses^11,18–21^. Despite significant progress in the Arabidopsis DHN field, their molecular action mode remains elusive.

In our previous research we found that the over-expression of the OpsDHN1 from *Opuntia streptacantha* improved cold tolerance in *A*. *thaliana* ^7^; at the protein level the OpsDHN1 is able to interact with itself in both *in vitro* and *in vivo* systems^22,23^. With these data, the question that arose was: is this DHN/DHN interaction an isolated property or could other DHNs self-associate or interact with other DHNs *in planta*? Our main goal in this study was to investigate the homo- and heterodimerization of three representative members of the Arabidopsis basic and acidic groups (AtCOR47, AtERD10, and AtRAB18) through the Bimolecular Fluorescence Complementation technique (BiFC); as supporting data the *in vivo* subcellular localization of AtCOR47, AtERD10, and AtRAB18 DHNs were analyzed using GFP translational fusions. Here, we provide detailed evidence on the *in planta* Arabidopsis DHNs oligomeric complexes, and also about the interactions of AtCOR47, AtERD10, and AtRAB18 proteins with its acidic OpsDHN1 ortholog. We discuss the dimerization among DHNs as a distinctive characteristic of DHNs that could enhance their functions in plants under stress conditions.

## Results

### Subcellular localization of the Arabidopsis acids AtCOR47, AtERD10, and the basic AtRAB18 dehydrins

In order to visualize the *in planta* subcellular distribution of the *A*. *thaliana* AtCOR47, AtERD10 and AtRAB18 DHNs, we independently fused the DHN coding regions to GFP in the pMDC43 vector (Supplementary Figure 1A–D). Nuclear staining and fluorescence images acquisition were performed as described in the Material and Methods section. As a localization control, the *N*. *benthamiana* leaves were transformed with the pMDC43 vector (Supplementary Figure 1A and E). The confocal microscopy analysis of the AtCOR47 and AtERD10 acidic DHNs revealed fluorescent signal only in the cytosolic areas, indicating that these acidic proteins are excluded from the plant nuclei (Supplementary Figure 1F and G). These data are in agreement with our *in silico* localization prediction data of both acidic DHNs (http://abi.inf.uni-tuebingen.de/Services/YLoc/webloc.cgi)^24^, which indicated a probability of 72.5% for cytosolic localization for AtCOR47 and 96.7% probability for AtERD10 (data not shown). In contrast, the confocal analysis of the basic AtRAB18 DHN revealed a dual nuclear/cytosolic distribution for AtRAB18 *in planta* (Supplementary Figure 1H). According to our *in silico* prediction, there is a 62% probability that the AtRAB18 protein will be localized in the cytoplasm, and 38% probability that it will be placed in the nucleus (http://abi.inf.uni-tuebingen.de/Services/YLoc/webloc.cgi; data not shown)^24^.

### *In planta* AtCOR47 and AtERD10 homodimer formation

**Figure 1:**
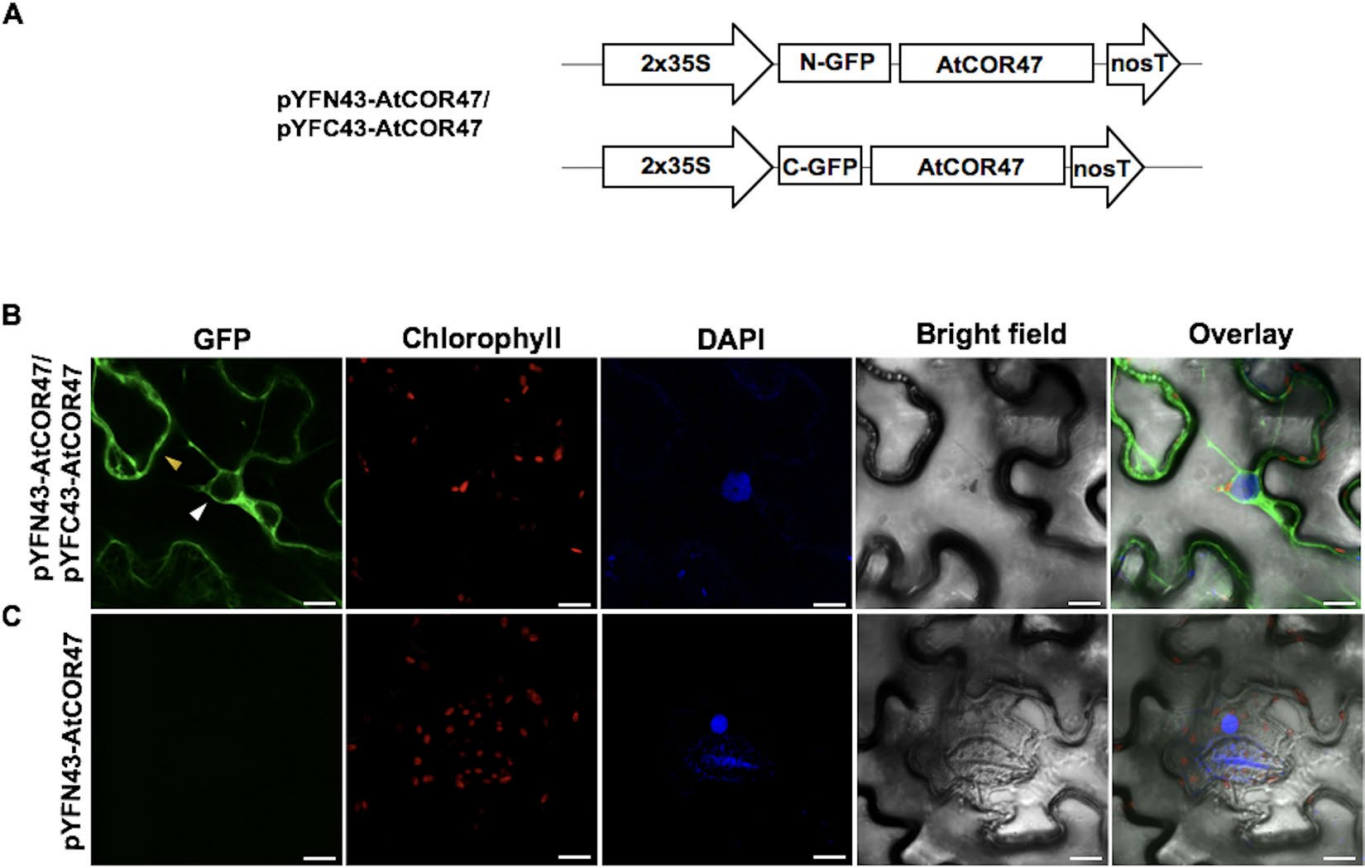
Determination of the AtCOR47 homodimers using the BiFC assay. (**A**) Schematic diagram of pYFN43-AtCOR47 and pYFC43-AtCOR47 BiFC vectors. (**B**) Confocal images of AtCOR47/AtCOR47 interaction. (**C**) Auto-fluorescence test in the BiFC system of the pYFN43-AtCOR47 construct transformed alone. From left to right: GFP, chlorophyll, DAPI, bright field, and overlay channels, white arrow indicates the nuclei and yellow arrow indicates the cytosol. The scale bar corresponds to 23 μm.

**Figure 2:**
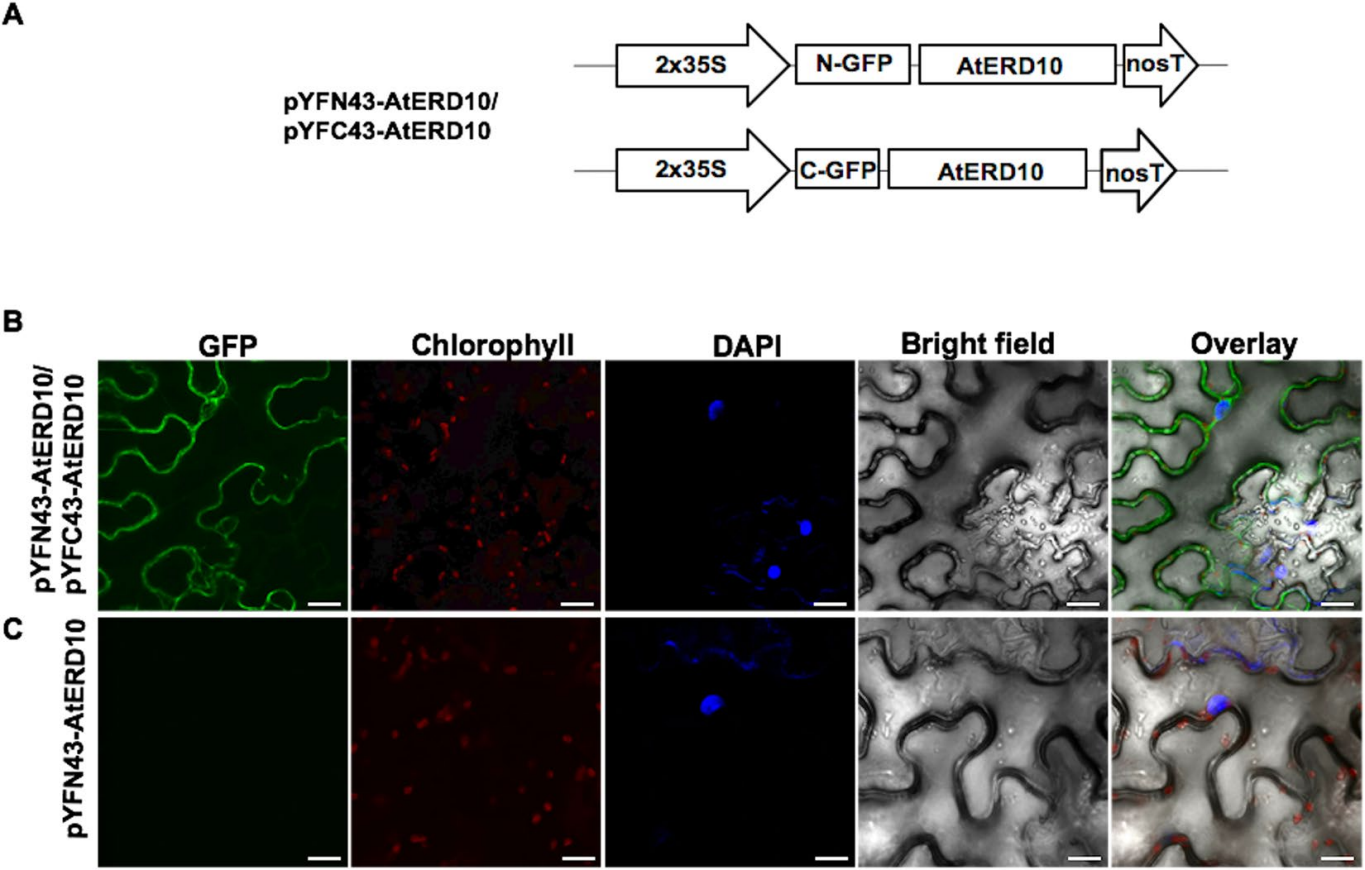
Visualization of the AtERD10 homodimers using the BiFC assay. (**A**) Schematic representation of BiFC vectors containing the AtERD10 protein fused to N- or C-terminus of GFP. (**B**) Fluorescence analysis of AtERD10/AtERD10 interaction. (**C**) Analysis for no fluorescent auto-activation of the pYFN43-AtERD10 construct. From left to right: GFP, chlorophyll, DAPI, bright field and overlay channels, white arrow indicates the nuclei and yellow indicates the cytosol. The scale bar corresponds to 23 μm.

Previously, we reported the self-association of the OpsDHN1 *in planta* ^23^. To explore whether other acidic DHNs, such as AtERD10 and AtCOR47, also have the ability to homodimerize, the AtERD10 and AtCOR47 coding regions were cloned into the BiFC vectors (Figs 1A and 2A); the generated constructs were evaluated in tobacco epidermal leaf cells. As shown in Figs 1B and 2B, the AtCOR47/AtCOR47 and AtERD10/AtERD10 interactions exhibited a strong fluorescent signal in the cytosol, but not in the nuclei of the plant cells. Also, the possible auto-fluorescence signal of the pYFN-AtCOR47 or pYFN-AtERD10 constructs was analyzed, but no fluorescent signal was detected (Figs 1C and 2C). As a positive control, the interaction between AKIN10 and AKINβ2 kinase subunits from Arabidopsis was used (Supplementary Figure 2). Additionally, the AKIN10 and AKINβ2 subunits were used as non-dehydrin interaction controls with AtCOR47 and AtERD10 BiFC constructs, but no fluorescence signal was detected (Supplementary Figures 3 and 4). These results show that AtCOR47 and AtERD10 can self-interact in a specific way in the cytosol of plant cells.

### *In planta* AtRAB18 homodimer formation

**Figure 3:**
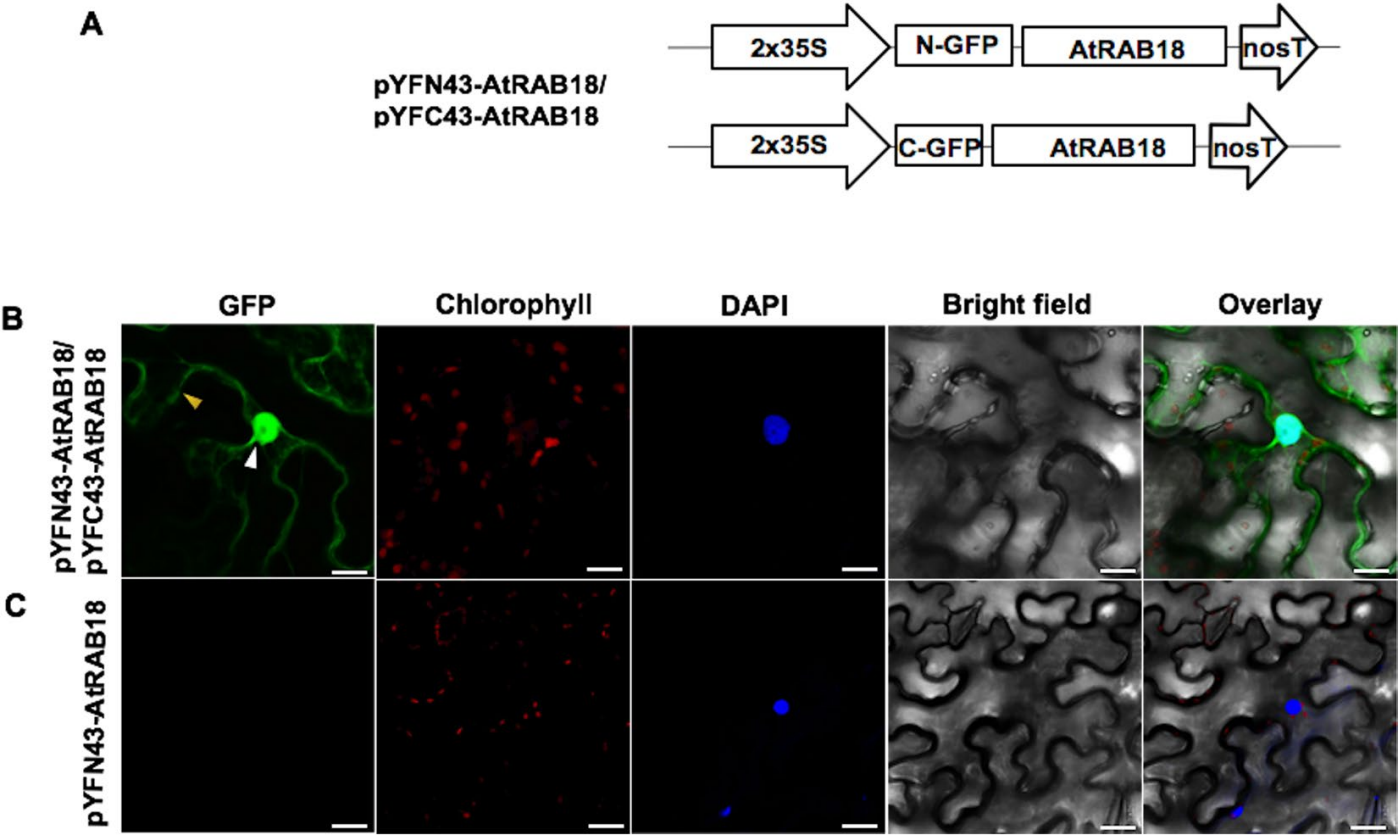
Detection of the basic AtRAB18 homodimer in BiFC assay. (**A**) Schematic representation of BiFC vectors containing the AtRAB18 protein fused to pYFN43 or pYFC43 vectors. (**B**) Fluorescence analysis of AtRAB18/AtRAB18 interaction. (**C**) Auto-fluorescence test of the pYFN43-AtRAB18 vector. From left to right: GFP, chlorophyll, DAPI, bright field and overlay panels. The white arrow indicates the nuclei and yellow arrow indicates the cytosol. The scale bar corresponds to 23 μm.

Our next step was to analyze whether the *A*. *thaliana* basic DHN AtRAB18 is capable of interacting in the BiFC system like their acidic paralogues do. The respective BiFC-AtRAB18 vectors were generated (Fig. 3A). In contrast to the acidic DHNs, the reconstitution of fluorescent signal mediated by AtRAB18 interaction was observed in the cytosol and nuclei of the tobacco epidermal cells (Fig. 3B). No fluorescent signal was detected when non-DHNs AKIN10 and AKINβ2 kinase subunits constructs were infiltrated in combination with the AtRAB18 BiFC constructs (Supplementary Figure 5), nor when the pYFN43-AtRAB18 was expressed alone (Fig. 3C). These data indicate that the basic AtRAB18 DHN interacts specifically with itself *in planta* in the cytosol and in the nucleus.

### *In planta* heterodimeric interactions between the Arabidopsis acidic DHNs AtCOR47, AtERD10 and the basic DHN AtRAB18

**Figure 4:**
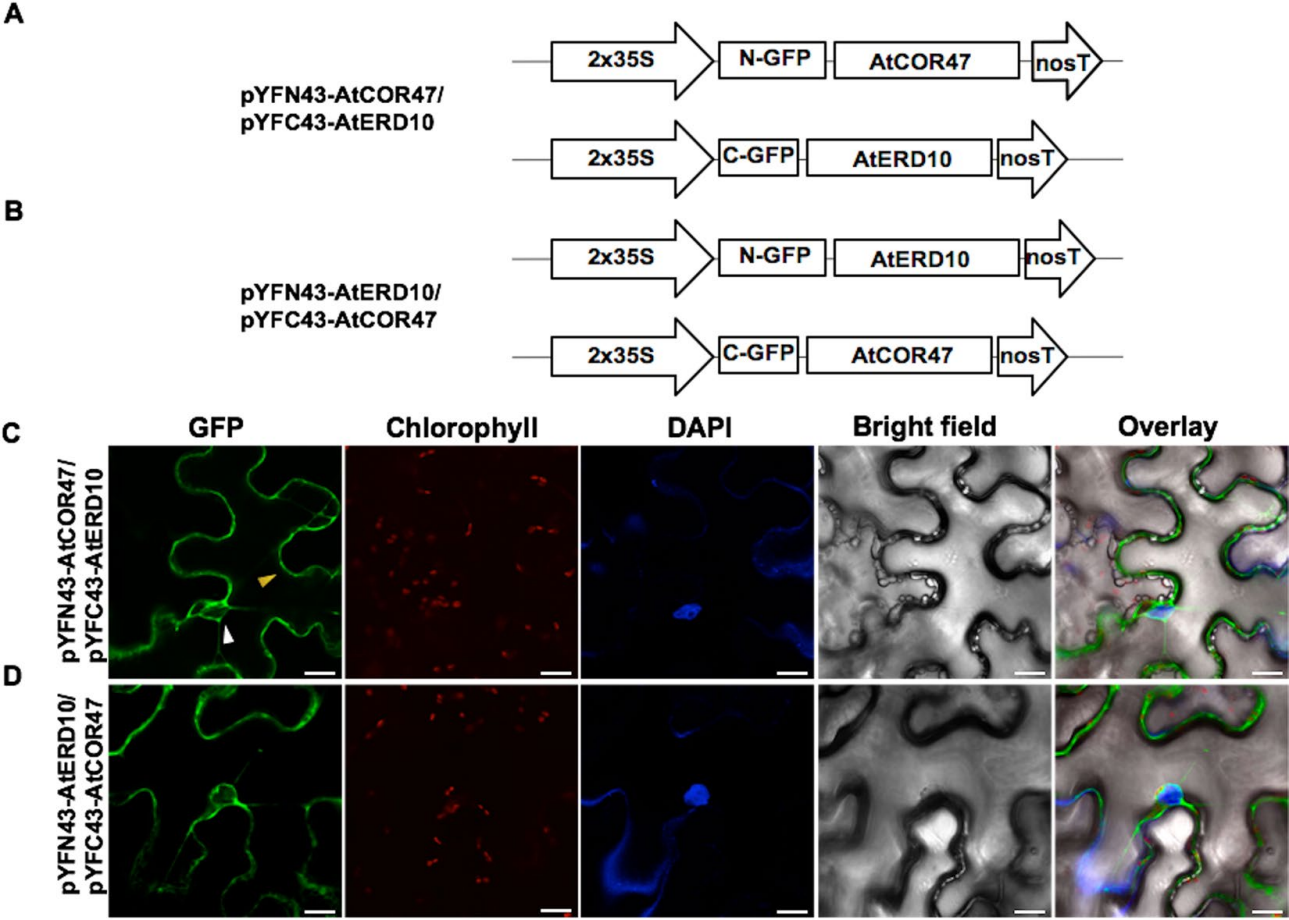
The AtCOR47 and AtERD10 DHNs are able to heterodimerize in BiFC assay. Schematic representation of the (**A**) pYFN43/pYFC43-AtCOR47 and (**B**) pYFN43/pYFC43-AtERD10 BiFC constructs. Confocal images of: (**C**) AtCOR47/AtERD10 and (**D**) AtERD10/AtCOR47 BiFC interactions. From left to right: GFP, chlorophyll, DAPI, bright field and overlay channels, white arrow indicates the nuclei and yellow arrow indicates the cytosol. The scale bar corresponds to 23 μm.

**Figure 5:**
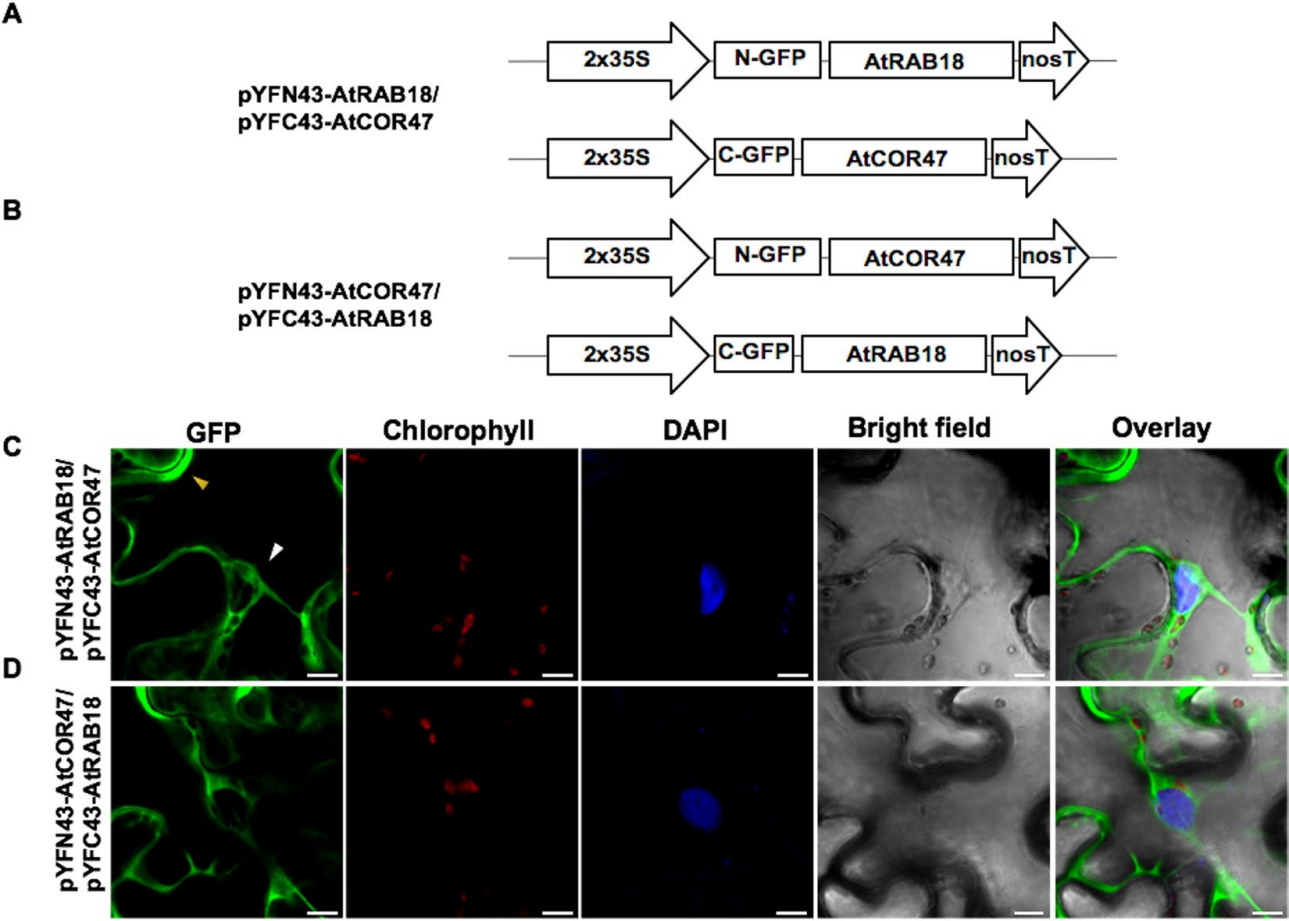
Heterodimerization between the Arabidopsis dehydrins: AtCOR47 and AtRAB18 in BiFC assay. (**A**) Schematic representation of the pYFN43-AtRAB18/pYFC43-AtCOR47 and (**B**) pYFN43-AtCOR47/pYFC43-AtRAB18 constructs. Fluorescent analyses of (**C**) AtRAB18/AtCOR47 and (**D**) AtCOR47/AtRAB18 interactions. From left to right: GFP, chlorophyll, DAPI, bright field and overlay channels, white arrow indicates the nuclei and yellow arrow targets the cytosol. The scale bar corresponds to 23 μm.

**Figure 6:**
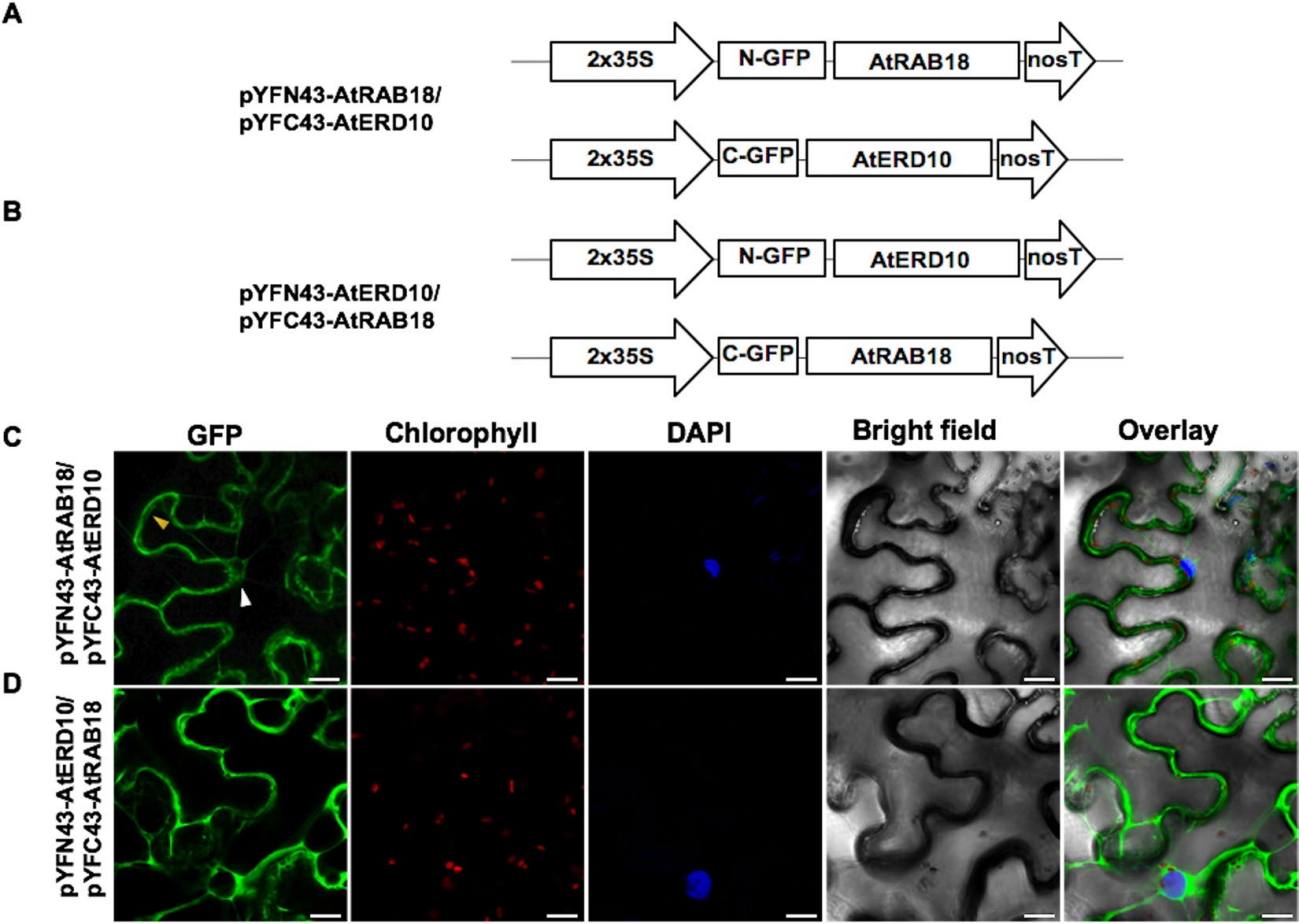
Identification of the AtERD10/AtRAB18 heterodimer interaction using the BiFC assay. Schematic illustration of the BiFC constructs containing the AtERD10 and AtRAB18 proteins. (**A**) The pYFN43-AtRAB18/pYFC43-AtERD10 constructs. (**B**) The pYFN43-AtERD10/pYFC43-AtRAB18 vectors. (**C**,**D**) Fluorescence visualization of AtRAB18/AtERD10 heterodimer formation. From left to right: GFP, chlorophyll, DAPI, bright field and overlay channels, white arrow indicates the nuclei and yellow arrows target the cytosol. The scale bar corresponds to 23 μm.

To investigate whether the Arabidopsis DHNs AtCOR47, AtERD10, and AtRAB18 can interact with each other, we performed the BiFC assay. For this analysis, AtCOR47, AtERD10, and AtRAB18 BiFC constructs were used (Figs 4–6, panels A and B). All constructs were co-expressed in all possible combinations in *N*. *benthamiana* epidermal leaf cells and analyzed for a fluorescent signal. As shown in Figs 4–6, panels C and D, the fluorescent signal is observed in all tested combinations (AtCOR47/AtERD10, AtRAB18/AtCOR47, and AtRAB18/AtERD10, or their swapped versions). The signals were confined to the cytosol of the tobacco plant cells, none of them colocalized with the DAPI nuclear signal. These results suggest that the formation of these heterodimers come from specific DHN/DHN interactions, and demonstrate that the interaction between the Arabidopsis DHNs is not only restricted to acidic group DHNs, since also the basic DHN AtRAB18 is capable of interacting with itself and with acidic DHNs AtCOR47 and AtERD10 *in planta*.

### Heterodimeric interactions of Arabidopsis DHNs AtCOR47, AtERD10, and AtRAB18 with their ortholog OpsDHN1

**Figure 7:**
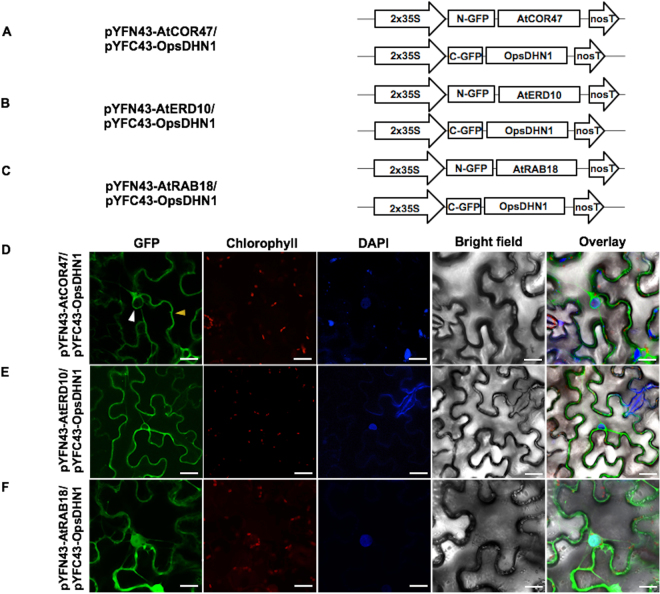
BiFC analysis of heterodimeric interaction among the Arabidopsis dehydrins: AtCOR47, AtERD10 and AtRAB18 with its orthologue OpsDHN1. Diagrammatic illustration of the BiFC constructs AtCOR47, AtERD10, AtRAB18 and OpsDHN1. (**A**) The pYFN43-AtCOR47/pYFC43-OpsDHN1 vectors. (**B**) The pYFN43-AtERD10/pYFC43-OpsDHN1 vectors. (**C**) The pYFN43-AtRAB18/pYFC43-OpsDHN1 vectors. Confocal interaction analysis of: (**D**) AtCOR47/OpsDHN1. (**E**) AtERD10/OpsDHN1. (**F**) AtRAB18/OpsDHN1. From left to right: GFP, chlorophyll, DAPI, bright field and overlay channels, white arrow indicates the nuclei and yellow arrows target the cytosol. The scale bar corresponds to 23 μm.

Previously, we demonstrated that the acidic OpsDHN1 from cactus pear forms dimers *in planta* ^23^. We conducted the BiFC assay to determine if the *A*. *thaliana* DHNs AtCOR47, AtERD10, and AtRAB18 can interact with their acidic ortholog OpsDHN1. For this, the OpsDHN1 BiFC construct^23^ and the AtCOR47, AtERD10, AtRAB18 BiFC constructs were employed (Fig. 7A–C). The plasmid co-expression, images acquisition and BiFC controls were performed as previously described. Our confocal analyses revealed fluorescent signal when the combinations AtCOR47/OpsDHN1, AtERD10/OpsDHN1, and AtRAB18/OpsDHN1 (Fig. 7D–F) or when their swapped versions were analyzed (Supplementary Figure 6). Acidic AtCOR47/OpsDHN1, AtERD10/OpsDHN1 heterodimeric complexes showed a restricted cytosolic distribution (Fig. 7D,E), no signal was detected inside the plant nuclei; however, the interaction between AtRAB18 and OpsDHN1 showed a dual nuclear/cytosolic distribution (Fig. 7F). Our data demonstrate that the three representative members of the Arabidopsis acidic and neutral groups (AtCOR47, AtERD10, and AtRAB18) are able to interact with their acidic OpsDHN1 ortholog *in planta*.

### The His-rich region present in the OpsDHN1 is not involved in DHN heterodimeric interactions

**Figure 8:**
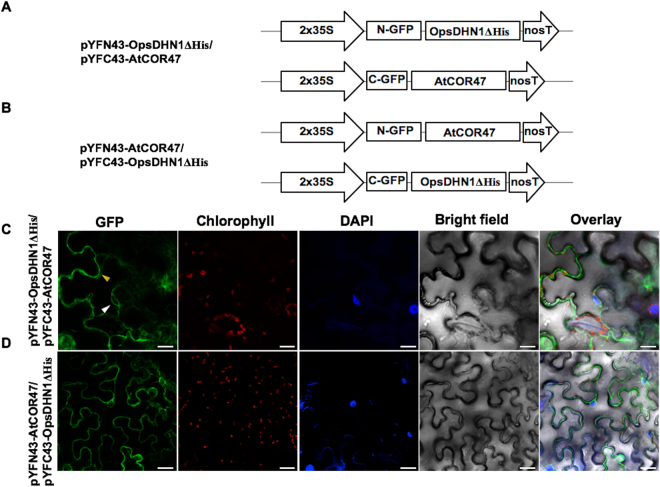
Analysis of interactions between OpsDHN1ΔHis version and AtCOR47 DHNs in BiFC system. Schematic depiction of: (**A**) pYFN43-OpsDHN1ΔHis/pYFC43AtCOR47. (**B**) pYFN43-AtCOR47/pYFC43-OpsDHN1ΔHis. (**C**,**D**) Confocal analysis of: OpsDHN1ΔHis/AtCOR47 interaction or its swapped version. From left to right: GFP, chlorophyll, DAPI, bright field and overlay channels, white arrow indicates the nuclei and yellow arrows target the cytosol. The scale bar corresponds to 23 μm.

**Figure 9:**
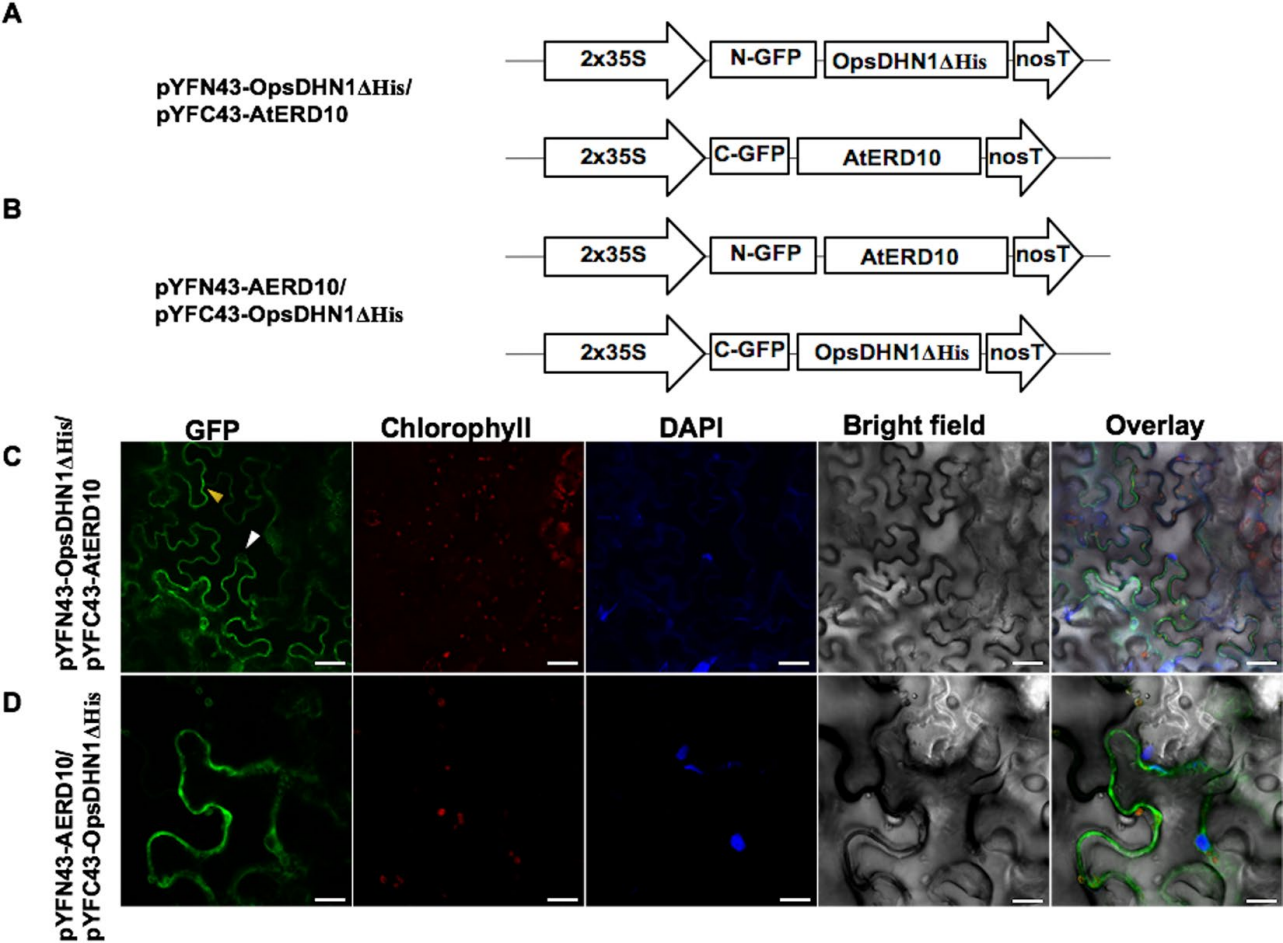
BiFC analysis of OpsDHN1ΔHis version and AtERD10 DHNs. Graphic representation of assayed constructs (**A**) pYFN43-OpsDHN1ΔHis/pYFC43-AtERD10 and (**B**) pYFN43-AtERD10/pYFC43-OpsDHN1ΔHis. Confocal pictures of: (**C**) OpsDHN1ΔHis/AtERD10 and (**D**) AtERD10/OpsDHN1ΔHis interactions. From left to right: GFP, chlorophyll, DAPI, bright field and overlay channels, white arrow indicates the nuclei and yellow arrows target the cytosol. The scale bar corresponds to 23 μm.

**Figure 10:**
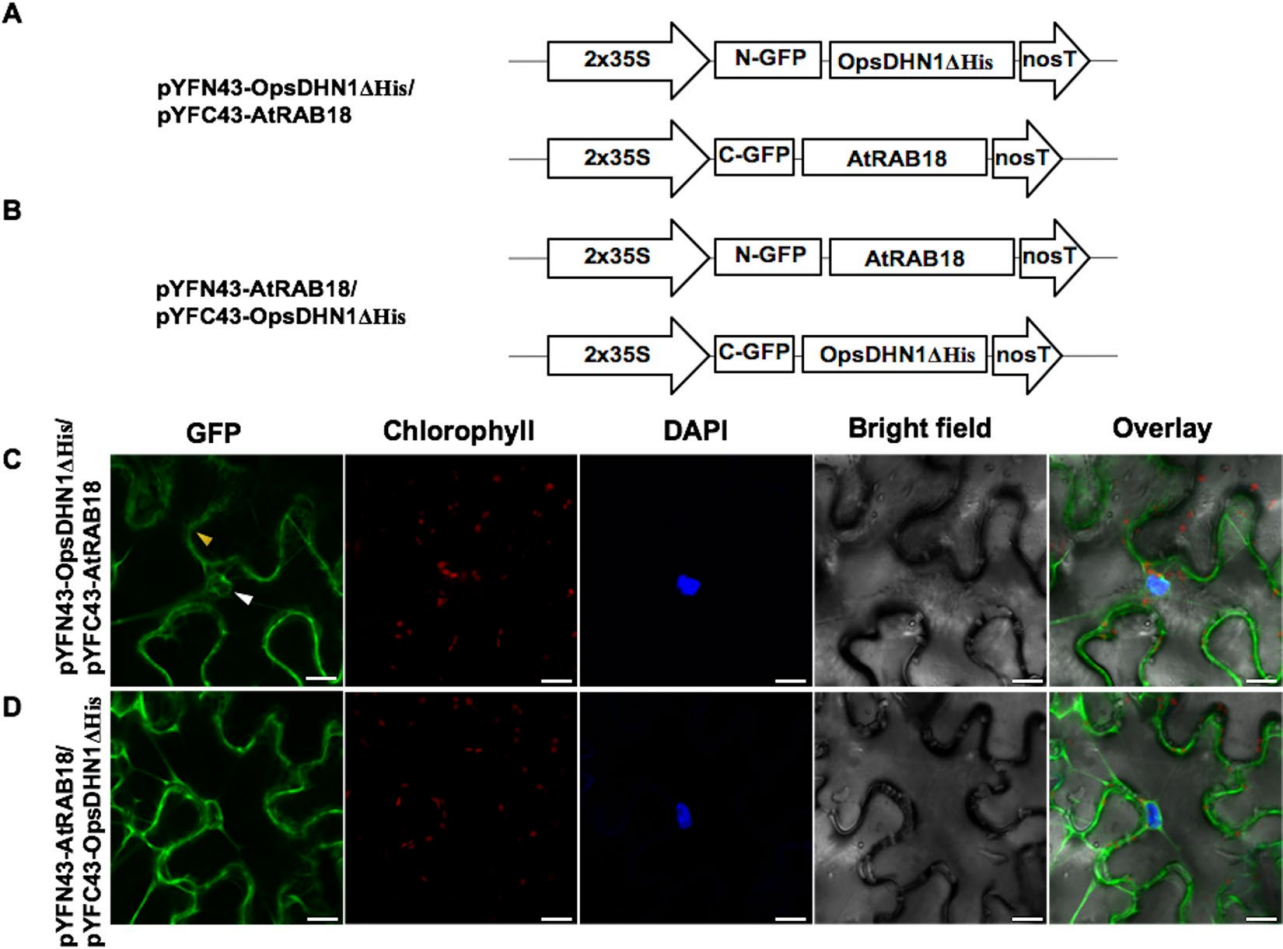
Interaction assay of OpsDHN1ΔHis version and AtRAB18 DHNs in BiFC system. A simplified representation of BiFC constructs. (**A**) pYFN43-OpsDHN1ΔHis/pYFC43-AtRAB18 and (**B**) pYFN43-AtRAB18/pYFC43-OpsDHN1ΔHis. Confocal pictures of transient co-transformed vectors. (**C**) OpsDHN1ΔHis/AtRAB18 and (**D**) AtRAB18/OpsDHN1ΔHis. From left to right: GFP, chlorophyll, DAPI, bright field and overlay channels, white arrow indicates the nuclei and yellow arrows target the cytosol. The scale bar corresponds to 23 μm.

To analyze whether the distinctive His-rich region present in the OpsDHN1 protein is involved in the interaction with AtCOR47, AtERD10 and AtRAB18, we co-transformed the OpsDHN1ΔHis version^22^ with Arabidopsis DHNs in tobacco leaf (Figs 8–10A and B). As shown in Figs 8 and 9 (C and D), the OpsDHN1ΔHis version was still able to interact with AtCOR47 and AtERD10 DHNs in the cytosol of plant cells. In the case of AtRAB18, we also detected fluorescence when AtRAB18 interacted with the OpsDHN1ΔHis version, but the localization of this heterodimer was exclusive to the cytosol (Fig. 10C, D), a behavior that we had previously observed in the OpsDHN1ΔHis/OpsDHN1-full version interaction^23^. These data demonstrated that the His-rich region is not required for the interaction of the OpsDHN1 with AtCOR47, AtERD10, and AtRAB18 DHNs *in planta*, and also reinforce our previous results where we showed that this segment is involved in the OpsDHN1 nuclear localization.

## Discussion

In a cellular context, proteins are often organized into dynamic groups to perform their biological functions^25^. Stress response is orchestrated at different levels through proteins that usually team up into oligomers. The accumulation of DHNs during abiotic stress has been reported in herbaceous dicotyledons, woody plants, and cereals^26^. In *Arabidopsis thaliana*, AtERD10, AtCOR47, and AtRAB18 DHNs contribute to the abiotic stress response; however, their *in vivo* molecular mechanism is unknown^18,21,26,27^. In the present work, we reported the formation of homodimeric complexes of Arabidopsis acidic and basic DHNs, and also discover the ability of DHNs to form heterodimers.

To increase our knowledge of these Arabidopsis proteins, we first aimed to determine the subcellular localization of the *A*. *thaliana* DHNs AtCOR47, AtERD10 and AtRAB18 using GFP translational fusions. Our data indicate that both acidic DHNs AtCOR47 and AtERD10 are located in the cytosol of *Nicotiana benthamiana* epidermal leaf cells. In the case of AtRAB18, this basic protein displays a dual nuclear/cytosolic localization; these results are in agreement with our *in silico* subcellular localization analysis (YLoc web server, data not shown)^24^. Studies performed by Candat *et al*.^28^ using GFP translational fusions reported that 36 out of 51 *A*. *thaliana* LEA members are cytosolic or cytosolic/nuclear proteins in Arabidopsis leaf protoplasts. Our results and these previous data are in agreement with AtCOR47 and AtERD10 *in vivo* cytosolic localization and with AtRAB18 nuclear/cytosolic localization. The tissue-specific localization of AtERD10 and AtRAB18 DHNs has been reported under normal and stress conditions^21^. The authors reported that during normal conditions the AtERD10 protein is localized in the root tips and vascular tissues of roots and stems, but no signal was detected in flowers and leaves, while, under stress conditions, the AtERD10 protein displayed a general distribution in roots and stems^21^. In contrast, the tissue distribution of AtRAB18 protein under normal conditions was observed in stems, leaves, flowers, and stomatal guard cells, but not in root cells. Under stress conditions a general immunohistochemical localization for AtERD10 and AtRAB18 proteins was observed in roots, stems, leaves and flowers tissues^21^. These data suggest a tissue general distribution of these DHNs under certain stress conditions^21^. According to this, a broad tissue localization of AtERD10 and AtRAB18 DHNs under stress conditions is not surprising, since immunohistochemical and fractionation studies have revealed the presence of other DHNs in almost all vegetative tissues and several cellular compartments, even in control conditions^29^. Although our data obtained with tobacco epidermal leaf cells are not representative of all cell types and tissues, it provides a reliable system to analyze the subcellular localization and intrinsic information encoded in DHN proteins. Resolving the AtCOR47, AtERD10 and AtRAB18 subcellular localizations could help to establish their particular biological functions inside a specific cellular compartment.

Recently, we reported the first *in planta* DHN association where the dimeric state of the OpsDHN1 acidic DHN from *Opuntia streptacantha* was discovered with a dual nuclear/cytosolic distribution^23^. To explore the *in vivo* homodimer formation in other plant species, we selected three representative members of the acidic and basic/neutral groups of the model plant *A*. *thaliana*. We first analyzed the homodimerization between the acidic DHNs AtCOR47 and AtERD10 through a BiFC assay. Our data revealed that both acidic AtCOR47 and AtERD10 proteins assemble into homodimers (AtCOR47/AtCOR47 and AtERD10/AtERD10) in the cytosol of Nicotiana leaves. In order to determine if homodimer formation is exclusive to acidic DHNs, the basic DHN AtRAB18 was also examined. We demonstrated that the Arabidopsis AtRAB18 DHN can also assemble into homodimers (AtRAB18/AtRAB18) in the nucleus and cytosol of the tobacco leaves.

**Figure 11:**
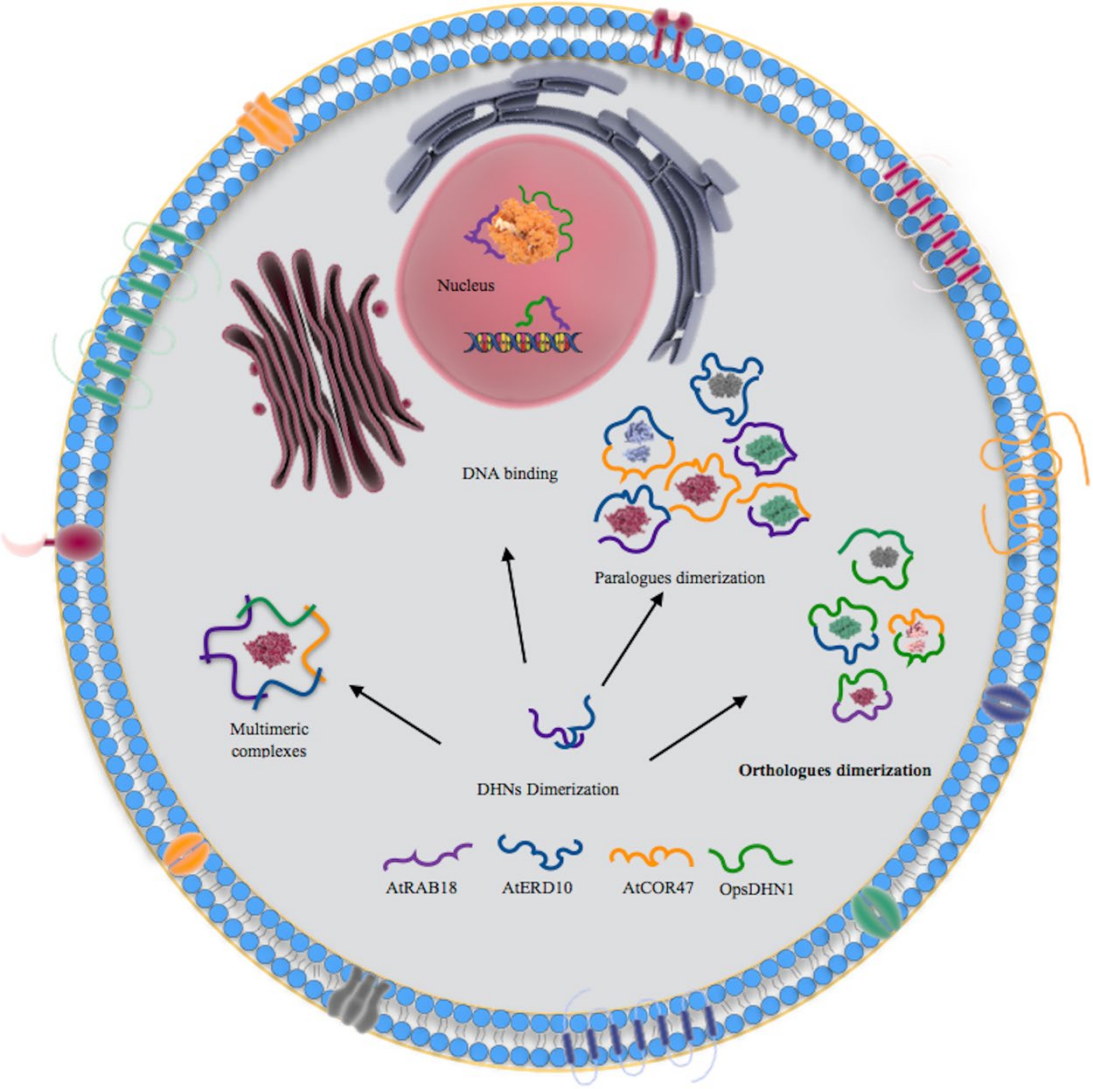
Proposed model for DHN dimerization in plant cells. Representation of homo- and heteromeric interaction among Arabidopsis dehydrins, and its interaction with the *Opuntia streptacantha* OpsDHN1 in tobacco cells. According to their localization results, the interactions AtCOR47/AtCOR47, AtCOR47/OpsDHN1 AtCOR47/AtERD10 and AtERD10/AtERD10, AtERD10/OpsDHN1 proteins only occur in the cytosol and could be protecting proteins from damage caused by stress. In contrast, AtRAB18/AtRAB18 and AtRAB18/OpsDHN1 were observed in both the cytosol and nucleus and could be protecting proteins from stress damage in either subcellular sites or by interacting with nucleic acids. This figure was created using Somersault 18:24 images as templates (http://www.somersault1824.com/science-illustrations/).

Interestingly, previous *in vitro* studies have suggested the formation of homo-oligomeric complexes for some DHNs such as the case of the homotetramer (350 kDa) of COR85 from *Espinacea olaracea* that had been purified from acclimated and non-acclimated plants^30^, and the homodimer (42 kDa) of the 20 kDa DHN from *Zea mays* purified from whole plant extracts^31^; in both studies the presence of DHN complexes was proven through the use of SDS-PAGE and gel filtration. Also, Still *et al*.^32^ demonstrated the accumulation of two DHN bands of 21 kDa and 38 kDa in Western blots of *Oriza sativa* embryo extracts 15 days after anthesis, suggesting that this 38 kDa band could be the dimeric form of the 21 kDa DHN. Recently, Rahman *et al*.^33^ using single-molecule force spectroscopy reported a potentially dimeric state for the recombinant TsDHN-2 from *Thellungiella salsuginea* ^33^. These data support the idea that Arabidopsis AtCOR47, AtERD10, and AtRAB18 dimerization is not an isolated event, instead pointing out an association as a common feature of DHNs (see Fig. 11).

Plant tolerance and survival is attained by the accumulation of a subset of stress-response proteins. In this respect, protein-protein interactions are a central part of this response, so one of the major challenges is to elucidate this network. Here, we tested the capacity of AtCOR47 and AtERD10 to form heterodimers in a BiFC system. Our results revealed that both Arabidopsis acidic DHNs are able to assemble into heterodimeric complexes (AtCOR47/AtERD10) *in planta* with a cytosolic distribution. These findings were extended to the acidic-basic DHN interaction, demonstrating heterodimeric formations between the acidic DHNs AtCOR47 and AtERD10 with the basic DHN AtRAB18 also occurs. Finally, we showed that the Arabidopsis acidic AtCOR47 and AtERD10 DHNs are capable of interacting with their *O*. *streptacantha* OpsDHN1 ortholog in the cytosol of Nicotiana leaf cells. Lastly, the basic DHN AtRAB18 was able to interact with the acidic OpsDHN1; however, this interaction was observed in the cytosol and nuclei of tobacco cells.

The ability of DHN to form *in planta* homo- and heterodimers interaction at specific subcellular locations could help to reinforce models proposed for DHN function. It is worthwhile to mention that the identity between the Arabidopsis acidic paralogues AtCOR47 and AtERD10 is 65%, and with their acidic OpsDHN1 orthologue is around 35%. However, the sequence identity between these acidic proteins with RAB18 is less than 19%, despite this, these proteins are capable of heterodimerizing. Our previous findings using the yeast two-hybrid system demonstrated that the conserved K-segments of OpsDHN1 contribute to the DHN/DHN interaction^22^. Here we also showed that the His-rich region does not take part in the interaction of OpsDHN1 with Arabidopsis DHNs AtCOR47, AtERD10, and AtRAB18. This data reinforces our previous findings, where we reported that this segment does not participate in OpsDHN1/OpsDHN1 dimerization, instead this particular segment is necessary for its nuclear localization^23^. Thus, we propose that, as in the case of the OpsDHN1 self-interaction, the conserved K-segments of DHNs could play a role in establishing the interaction, both in the formation of homo- and heterodimers. In spite of DHNs possessing a low proportion of hydrophobic amino acids, some of them, such as Ile and Leu, are conserved in the K-segments^34^. Several authors have demonstrated that the amphipathic K-segments can form a α-helix^2^. We hypothesized that the hydrophobic Ile and Leu amino acids may be contributing in the formation of hydrophobic patches on one side of K-segment helixes, and therefore could be involved in DHN/DHN interactions; however, more experiments are needed to provide evidence for this hypothesis.

The over-expression of a single DHN gene generally results in an improvement in plant stress tolerance^35–38^. However, this is not an absolute rule since in some cases the over-expression of a DHN gene resulted in a slight or even no measureable increase in stress tolerance^39–41^. Puhakainen *et al*.^12^ generated transgenic Arabidopsis lines over-expressing pairwise combinations of four DHNs. Despite all lines accumulating similar DHN levels, pTP9 (expressing AtRAB18 and AtCOR47) and pTP10 (harboring AtERD10 and LTI30) transgenic lines showed significant differences in freezing tolerance, 41% for TP9 lines and 86% for TP10 lines in comparison to 22% in control plants, but no freezing tolerance was obtained when a single DHN was over-expressed. We propose that the dimerization among DHNs could offer advantages since the association of two shorter proteins could be a more effective molecular shield than one, as determined by Hughes *et al*.^42^, where the authors observed that longer DHNs are more efficient at protecting LDH from activity loss *in vitro*. Also, DHNs, as intrinsically disordered proteins, are susceptible to random degradation, so this interaction could favor avoiding proteolysis^43,44^. The accumulation of different types of DHNs in low temperature or ABA-treated plants suggests that the role of DHNs is accentuated during conditions of stress^21^, and also that different DHN combinations could determinate the behavior and function of these proteins in response to stress stimuli.

Quaternary associations have also been described, at least *in vitro*, for several other LEA proteins. Members of the LEA 3 group are predicted to exist as dimers, interacting principally through amphipathic α-helices formed by their 11-mer motifs^9,45^. A mass spectrometric approach revealed that the AfrLEA2 LEA 3 group could exist as homodimers and homotrimers in embryos of *Artemia franciscana* ^46^, and Goyal *et al*.^47^ used immunoblotting and cross-linking experiments to demonstrate that AavLEA1 from *Aphelenchus avenae* (LEA group 3) is present as oligomers in solution. Interestingly, it has been proposed that LEA group 2 (DHNs) and group 3 are phylogenetically closely related^9^.

This observation extends to other LEA proteins. High molecular mass complexes of AtLEA4-2 (LEA group 4) were obtained from cell extracts of *A*. *thaliana* stressed plants^48^. Rivera-Najera *et al*.^49^ also saw *in vivo* dimerization of PvLEA6 (LEA group 6) from *Phaseolus vulgaris* using the BiFC assay; using the same approach, the dimerization of RcLEA (LEA group 7) from *Rosa chinensis* was seen in Nicotiana leaves^50^. Based on previous findings of high molecular mass complexes detected in cell extracts from stressed plants, Olvera-Carrillo *et al*.^51^ proposed that the interaction could occur across the LEA family, and that the conserved sequences present in the different LEA groups could play a role in the formation of high order structures between LEA proteins from the same or different groups. Our data on homo- and heterodimeric interactions among AtCOR47, AtERD10, and AtRAB18 constitute the first report on Arabidopsis DHN associations in plant, however, using the BiFC assay we can only analyze dimerization between two proteins, and cannot discard the possibility of the formation of tetramers or other higher order DHN oligomers without further experimentation.

The study of DHN interactions and subcellular localization is central to understanding the molecular mechanism by which these proteins work; the molecular shield effect^10,42,52^ and the disordered chaperone effect^53^ are the two models proposed for DHN function with respect to protecting proteins from abiotic stress damage. In both scenarios, DHN assembly into homo- and heterodimers could offer a combinatorial effect of producing multiple complexes with different affinities and specificities for their biological targets, giving the cell an instrument for fine-tuning its stress response.

## Material and Methods

### Plant material and growth conditions

*Nicotiana benthamiana* seeds were spread on a mix of 50% vermiculite and 50% soil, and grown in a greenhouse with long-day photoperiod cycles (16 h light/8 h dark) at 22 °C ± 2 °C for three to four weeks.

### Vector construction

First, the entry clones were generated by PCR amplification of each *AtCOR47* (At1G20440), *AtERD10* (At1G20450), and *AtRAB18* (At5G66400) open reading frame. Next, the PCR products were cloned into the pCR8/GW/TOPO TA Cloning entry vector (Invitrogen, Carlsbad, CA). Selected clones were sequenced using the M13 forward primer. Second, for subcellular analysis, GFP translational fusions were carried out through a recombination of the pCR8-AtCOR47, pCR8-AtERD10 and pCR8-AtRAB18 entry vectors with the pMDC43 binary destination vector^54^. To perform the Bimolecular Fluorescence Complementation (BiFC) test, the AtCOR47, AtERD10, and AtRAB18 entry clones were shifted into both pYFN43 and pYFC43 BiFC expression vectors^55^. All shift reactions were done by site-specific recombination using the Gateway LR Clonase II Enzyme Mix (Invitrogen). Finally, all generated destination vectors were introduced into *Agrobacterium tumefaciens* GV3101/pMP90 strains. The *A*. *tumefaciens* cells harboring the pYFN43-OpsDHN1 and pYFC43-OpsDHN1 vectors had been created previously^23^. BiFC interaction control vectors were donated by the Laboratory of Dr. A. Ferrando^55^.

### *Nicotiana benthamiana* transient transformation

Abaxial leaf cells from *N*. *benthamiana* plants were transiently transformed by *A*. *tumefaciens* GV3101/pMP90 strains containing the generated expression vectors. To inhibit gene silencing during the BiFC tests, an *A*. *tumefaciens* strain harboring the tomato bushy stunt virus p19 protein was used during co-infiltration^55,56^. The *A*. *tumefaciens* cells were collected at OD_600_ of 1.0, and resuspended in infiltration buffer (10 mM MgCl_2_, 10 mM MES pH 5.6, and 200 μM acetosyringone). Then the strains were incubated at room temperature on a rocking platform for 3 h. Afterward, the *N*. *benthamiana* leaf abaxial space was co-infiltrated using a needleless syringe. Three days after infiltration, two leaves from three independently transformed plants were analyzed for fluorescence under a confocal microscope. Analyses were performing by triplicate for all constructs, and gave similar results.

### Nuclei staining

For nuclei staining, DAPI reagent (Sigma, St. Louis, MO) was used. Briefly, *N*. *benthamiana* transformed leaf segments were cut from the plant and then incubated for 5 min in a water solution containing 5 μg/mL of DAPI.

### Fluorescence confocal microscopy

The transiently transformed *N*. *benthamiana* leaves were observed under a Leica TCS SP5 multiphoton confocal microscope (Leica, Wetzlar, Germany). The laser excitation wavelength was 488 nm and the spectral detection was set between 497–537 nm for GFP and 684–758 nm for chlorophyll fluorescence, with a beam splitter MBS 488. For DAPI laser excitation the wavelength was set to 405 nm and detection was made at 410–492 nm. The objective used was 20x Multi-Immersion. Image analysis was performed with Fiji imaging software^57^.

## Electronic supplementary material

Supplementary information
