# Supplementary material for: In vivo evidence for homo- and heterodimeric interactions of Arabidopsis thaliana dehydrins AtCOR47, AtERD10, and AtRAB18
Source: Sci Rep. 2017 Dec 6;7:17036. doi: 10.1038/s41598-017-15986-2 (PMC5719087; doi:10.1038/s41598-017-15986-2)
Supplement: Supplementary file 1 — Supplementary information [file 41598_2017_15986_MOESM1_ESM.pdf]

In vivo evidence for homo- and heterodimeric interactions of _Arabidopsis thaliana_ dehydrins COR47, ERD10, and RAB18

*Itzell E. Hernández-Sánchez[1], Israel Maruri-López[1], Steffen P. Graether[2], and Juan F. Jiménez-Bremont[1]**

[1]*Laboratorio de Biología Molecular de Hongos y Plantas, División de Biología Molecular, Instituto Potosino de Investigación Científica y Tecnológica AC, San Luis Potosí, México,*

[2]*Department of Molecular and Cellular Biology, University of Guelph, Guelph, ON, Canada.*

**Corresponding author: jbremont@ipicyt.edu.mx; jbremont@yahoo.com (J. F. Jiménez-Bremont)*

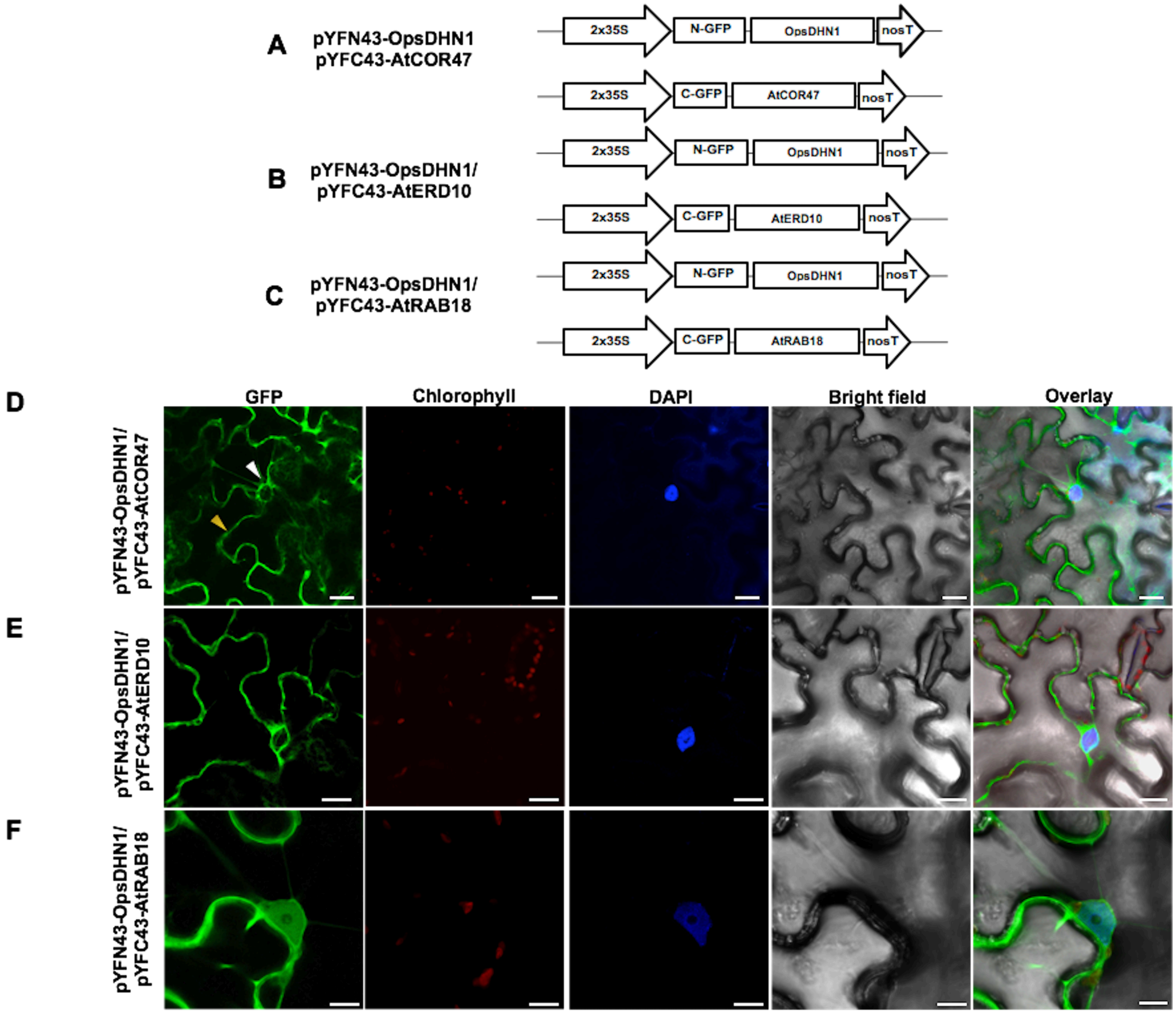

Supplementary Figure 1. *In vivo* subcellular localization of *A. thaliana* acidic AtCOR47, AtERD10 and the basic AtRAB18 DHNs. Schematic representation of the (A) pMDC43 vector and constructs containing (B) AtCOR47 (C) AtERD10, and (D) AtRAB18; all constructs are under the 2x35S promoter and nosT terminator. Subcellular localization of (E) control vector, and protein fusions (F) pMDC43-AtCOR47, (G) pMDC43-AtERD10, and (H) pMDC43-AtRAB18 in tobacco epidermal leaf cells. GFP is under the control of the 2x35S promoter and NosT terminator. From left to right: GFP, chlorophyll, DAPI, bright field, and overlay panels. The white arrow indicates the nuclei and yellow one indicates the cytosol. The scale bar corresponds to 23 μm.

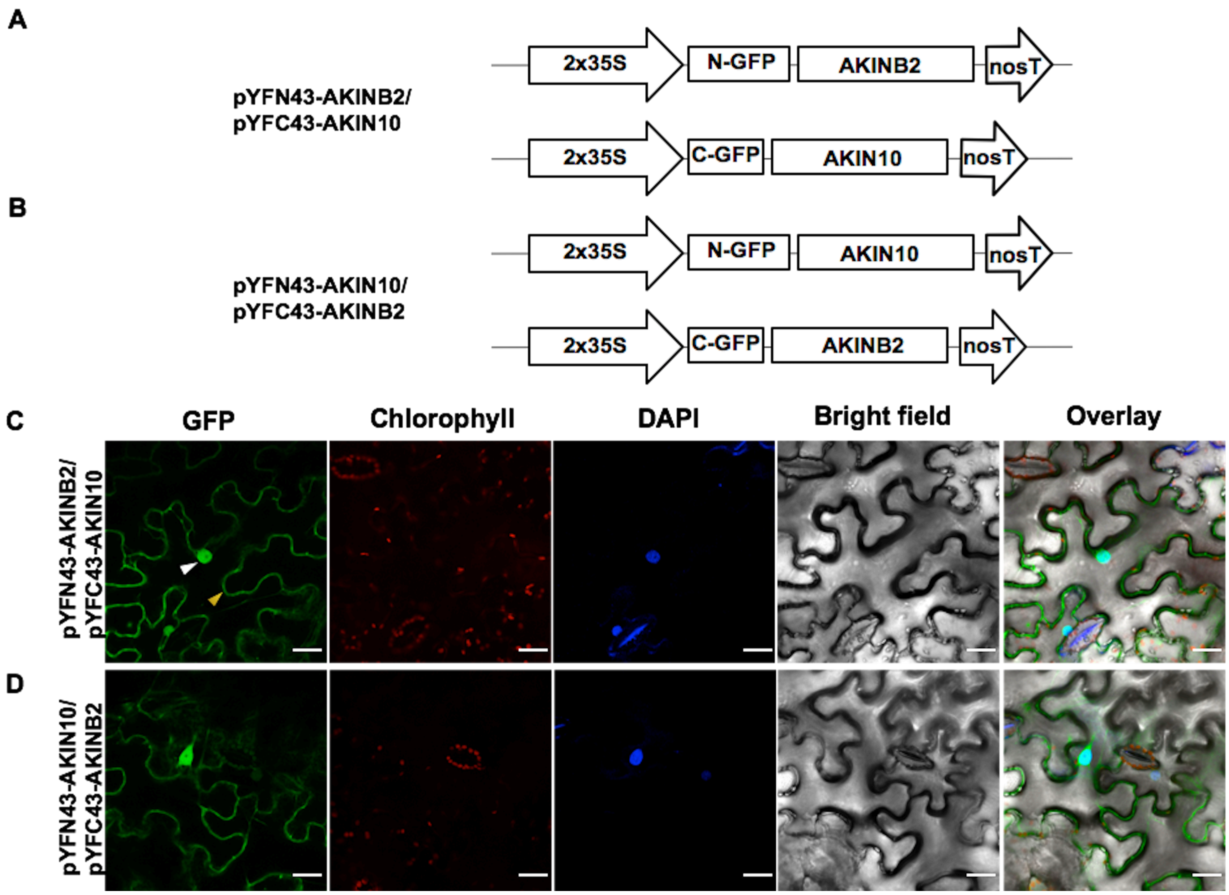

Supplementary Figure 2. Positive BiFC interaction controls. Schematic representation of BiFC vectors containing the *A. thaliana* SnRK kinases AKIN10 and AKINβ2 fused to N- or C-terminus of GFP. (A) The pYFN43-AKINβ2 and pYFC43-AKIN10 vectors. (B) The pYFN43-AKIN10 and pYFC43-AKINβ2 vectors. (C) Fluorescent analysis of pYFN43-AKIN10 and pYFC43-AKINβ2 and their swapped versions (D). From left to right: GFP, chlorophyll, DAPI, bright field and overlay channels, white arrow indicates the nuclei and yellow arrow indicates the cytosol. The scale bar corresponds to 23 μm.

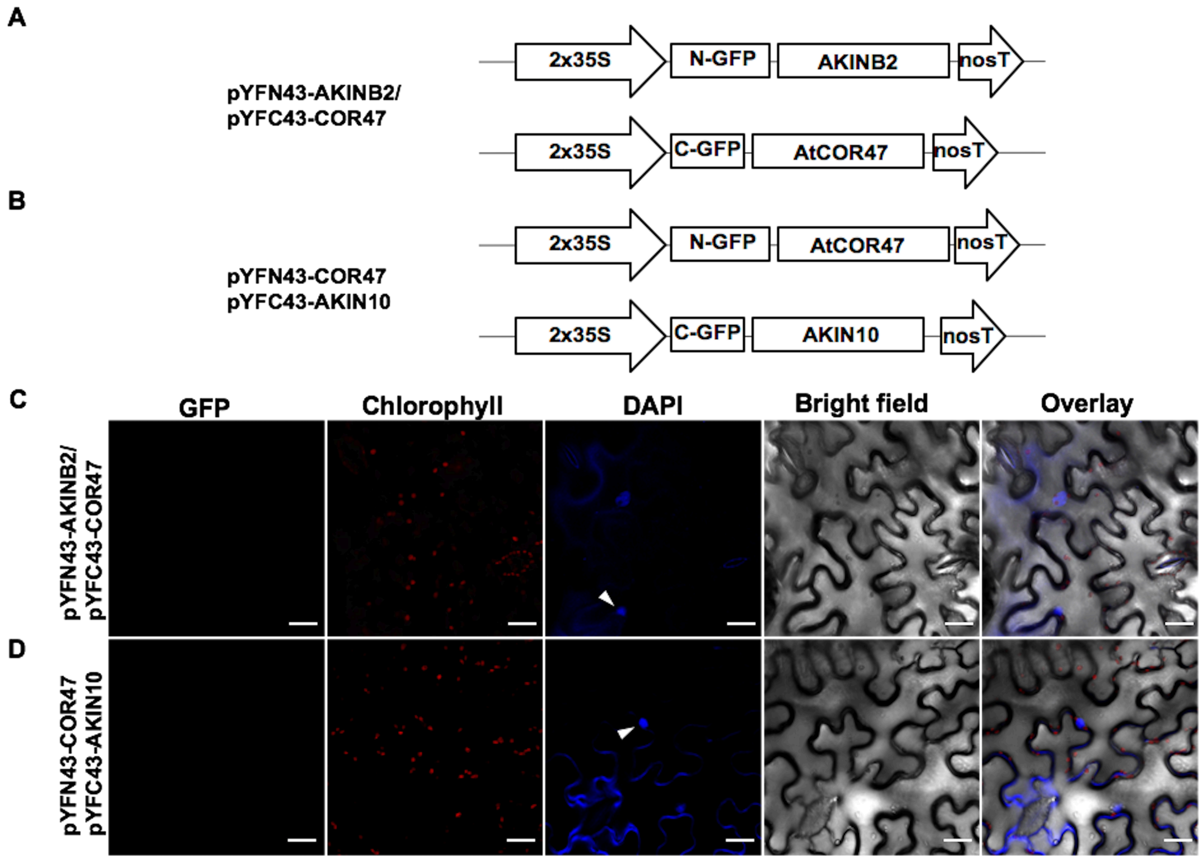

Supplementary Figure 3. Interaction analysis between AtCOR47 and non-DHNs proteins. Schematic representation of BiFC constructs containing either AtCOR47 or the *A. thaliana* SnRK kinases AKIN10 and AKINβ2 fused to the N- or C-terminus GFP. (A) Co-transformed combination of the pYFN43-AKINβ2/pYFC43-AtCOR47 constructs. (B) Co-transformed pYFN43-AtCOR47/pYFC43-AKIN10 vectors. (C) Fluorescent analysis of AKINβ2/AtCOR47 and (D) AtCOR47/AKIN10. Each combination was transiently co-transformed in *N. benthamiana* epidermal cells. From left to right: GFP, chlorophyll, DAPI, bright field and overlay channels, white arrow indicates the nuclei. The scale bar corresponds to 23 μm.

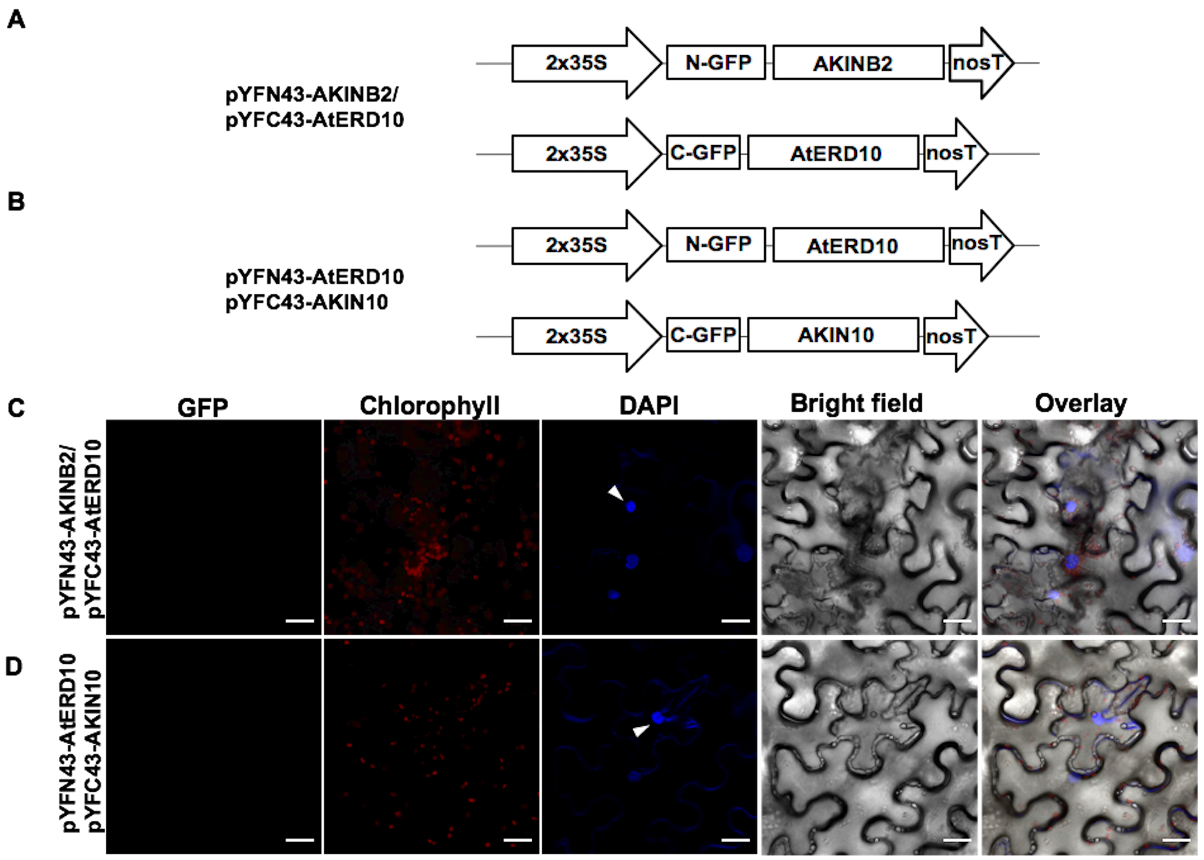

Supplementary Figure 4. Interaction analysis between AtERD10 and non-DHNs proteins. Schematic representation of BiFC vectors containing either AtERD10 or the *A. thaliana* SnRK kinases AKIN10 and AKINβ2 fused to the N- or C-terminus of GFP. (A) Co-transformed combination of the pYFN43-AKINβ2/pYFC43-AtERD10 constructs. (B) Co-transformed pYFN43-ERD10/pYFC43-AKIN10 vectors. (C) Fluorescent analysis of AKINβ2/AtERD10 and AtERD10/AKIN10, each combination was transient co-transformed in *N. benthamiana* epidermal cells. From left to right: GFP, chlorophyll, DAPI, bright field and overlay channels, white arrow indicates the nuclei. The scale bar corresponds to 23 μm.

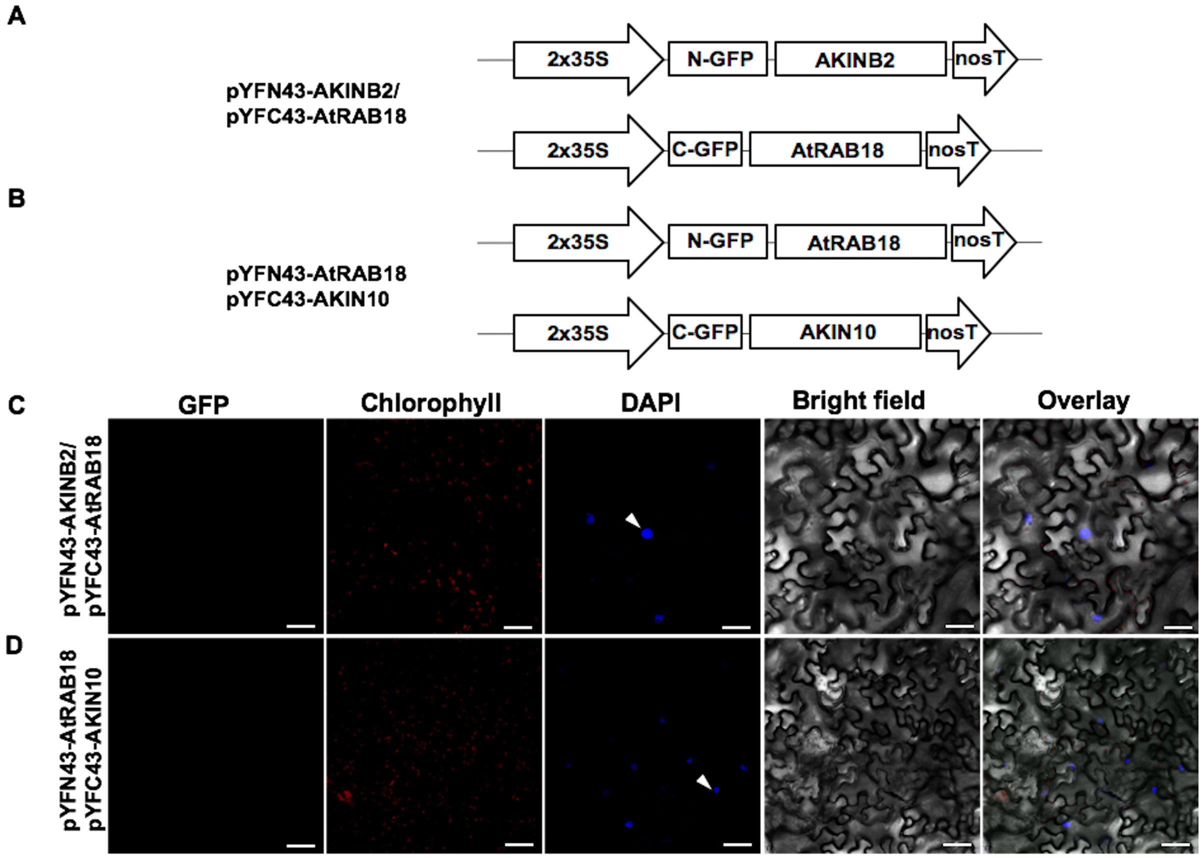

Supplementary Figure 5. Interaction analysis between AtRAB18 and non-DHNs proteins. Schematic representation of BiFC vectors containing either AtRAB18 or the *A. thaliana* SnRK kinases AKIN10 and AKINβ2 fused to the N- or C-terminus of GFP. (A) Co-transformed combination of the pYFN43-AKINβ2/pYFC43-AtRAB18 constructs. (B) Co-transformed pYFN43-ERD10/pYFC43-AKIN10 vectors. (C) Fluorescent analysis of AKINβ2/AtRAB18 and AtRAB18/AKIN10, each combination was transient co-transformed in *N. benthamiana* epidermal cells. From left to right: GFP, chlorophyll, DAPI, bright field and overlay channels, white arrow indicates the nuclei. The scale bar corresponds to 23 μm.

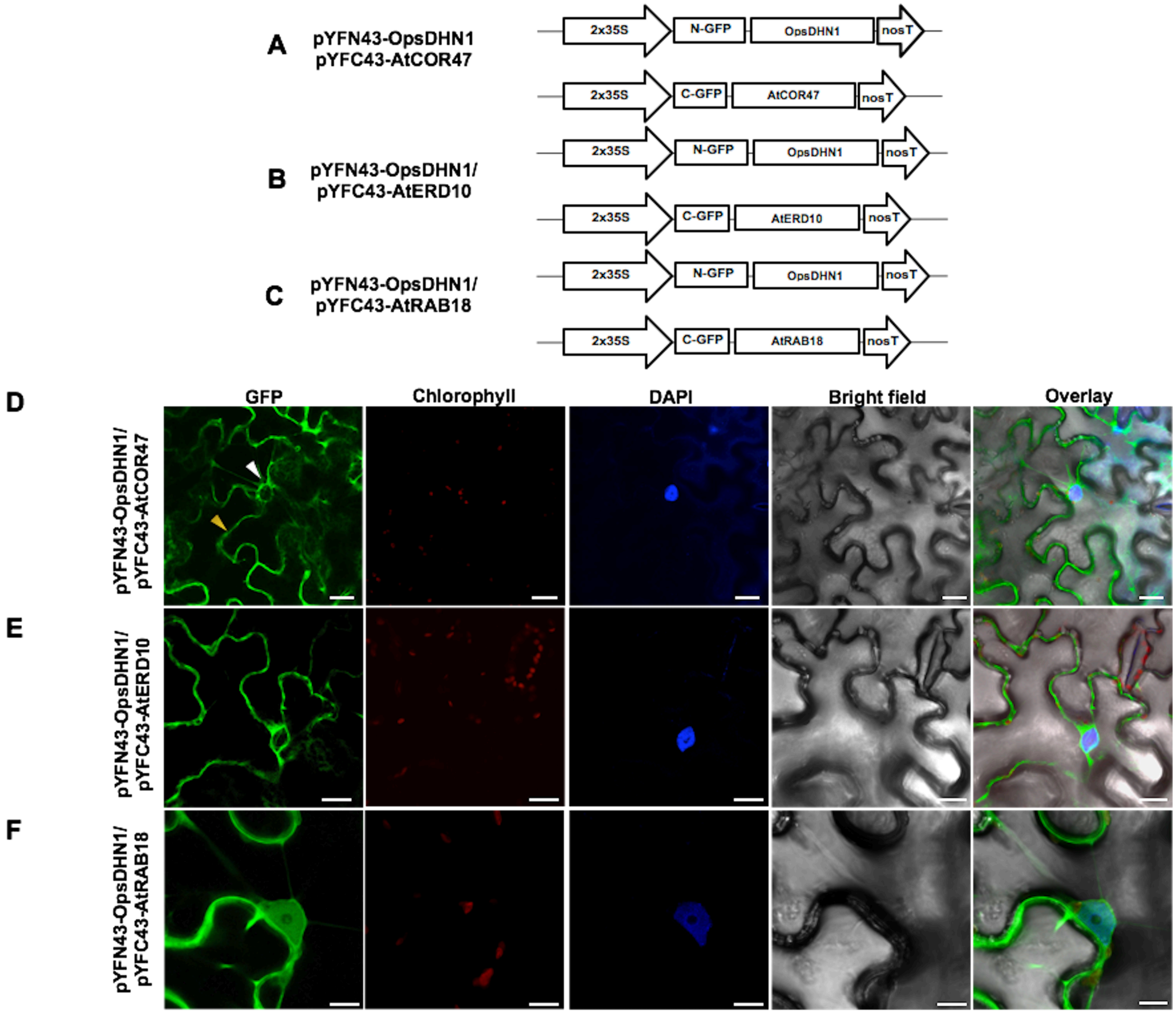

Supplementary Figure 6. Heterodimeric interactions between the swapped version of Arabidopsis dehydrins AtCOR47, AtERD10, and AtRAB18 with its orthologue OpsDHN1 from cactus pear. Diagrammatic illustration of the BiFC constructs AtCOR47, AtERD10, AtRAB18 and OpsDHN1 DHNs assayed for heterodimeric interactions. (A) The pYFN43-OpsDHN1/pYFC43-AtCOR47 vectors combination. (B) The pYFN43-OpsDHN1/pYFC43-AtERD10 vectors combination. (C) The pYFN43-OpsDHN1/pYFC43-AtRAB18 vectors combination. Confocal interaction analysis of the transient co-transformed (D) OpsDHN1/ AtCOR47 constructs (E) OpsDHN1/AtERD10 constructs and (F) OpsDHN1/AtRAB18 constructs. From left to right: GFP, chlorophyll, DAPI, bright field and overlay channels, white

arrow indicates the nuclei and yellow arrows target the cytosol. The scale bar corresponds to 23 μm.
